# Mucinous non neoplastic cyst of the pancreas: a case report

**DOI:** 10.1093/jscr/rjad633

**Published:** 2023-11-21

**Authors:** Ismail Elahabadi, Amir Rahnama, Gholamreza Bazmandegan, Zahra Kamiab

**Affiliations:** Department of Surgery, Ali Ibn Abi Talib Hospital, School of Medicine, Rafsanjan University of Medical Sciences, Imam Ali Boulevard, Rafsanjan 7717933777, Iran; Department of Pathology, School of Medicine, Rafsanjan University of Medical Sciences, Imam Ali Boulevard, Rafsanjan 7717933777, Iran; Physiology-Pharmacology Research Center, Research Institute of Basic Medical Sciences, Rafsanjan University of Medical Sciences, Imam Ali Boulevard, Rafsanjan 7717933777, Iran; Department of Physiology and Pharmacology, School of Medicine, Rafsanjan University of Medical Sciences, Imam Ali Boulevard, Rafsanjan 7717933777, Iran; Department of Physiology and Pharmacology, School of Medicine, Rafsanjan University of Medical Sciences, Imam Ali Boulevard, Rafsanjan 7717933777, Iran

**Keywords:** pancreatic cyst, mucinous nonneoplastic cyst (MNNC), ovarian-like stroma

## Abstract

The aim of this study was to introduce a patient with mucinous nonneoplastic cyst (MNNC) at an unusual age. MNNCs of the pancreas are uncommon primary tumors, which affect middle-aged women in their fifth decade of life and have significant malignant potential. Therefore, it is important to accurately diagnose and remove them. This case is a 28-year-old woman patient who presented with a pain in the right and upper abdomen from 3 months ago and worsened at night. A cystic lesion was observed near the upper bridge of the left kidney in abdominal ultrasonography. Contrast-enhanced abdominal computed tomography (CT) scan showed a cystic lesion in the trunk and umbilical cord of the pancreas. The patient underwent surgery and the mass was removed and the MNNC was diagnosed.

## Introduction

Approximately 10% of cystic lesions of the pancreas are cystic neoplasms that make up 1% of all pancreatic tumors [1]. Cystic neoplasms may originate from the ductal epithelium [serous cystic neoplasms (SCN)], mucinous cystic neoplasms (MCN), intraductal papillary mucinous neoplasms (IPMN), or endocrine cells (intraductal tubular neoplasms) or from pancreatic acidosis and mesenchyme elements (cyst adenoma and cystadenocarcinoma) [2]. MCN are unusual primary tumors that usually affect middle-aged women in their fifth decade of life. Although these neoplasms are less common than SCNs, they have significant malignant potential [3]. Therefore, it is very important to identify and remove them. In the 2010 World Health Organization classification of tumors, MCN is defined as a cystic-structured epithelial neoplasm that does not communicate with the pancreatic duct and is composed of mucin-producing columnar epithelium. These cysts have pathological and clinical similarities with cystic tumors of the ovarian mucosa and biliary cystadenoma of the liver [4]. The aim of this study was to introduce a patient with MCN of unusual age and with nonspecific symptoms.

## Case report

The patient was a 28-year-old woman with complaints of a pain in her upper left abdomen, which she had referred on 22 October 2019. The pain in the patient had started 3 months earlier. The pattern of pain was that the pain was consistently low, but worsened at night. The patient had no symptoms of nausea and vomiting, no weight loss, no fever, no icterus, no history of surgery, no special medication, but antacid therapy had been used for her for about a month, but the pain had not changed. Her family background was negative. The patient’s vital signs and abdominal examination were normal (Table 1).

**Table 1:** Mucinous nonneoplastic cyst patient laboratory tests at the time of referral

WBC (x 106/L)	4300
HB (g/L)	11/2
MCV (fL)	78/37
PLT (x 106/L)	171
Bilirubin (mg/dL)	1/5
AST (U/L)	17
ALT (U/L)	7
ALP (IU/L)	165
Urea (mg/dL)	29
Creatinine (mg/dL)	0/8

**Figure 1:**
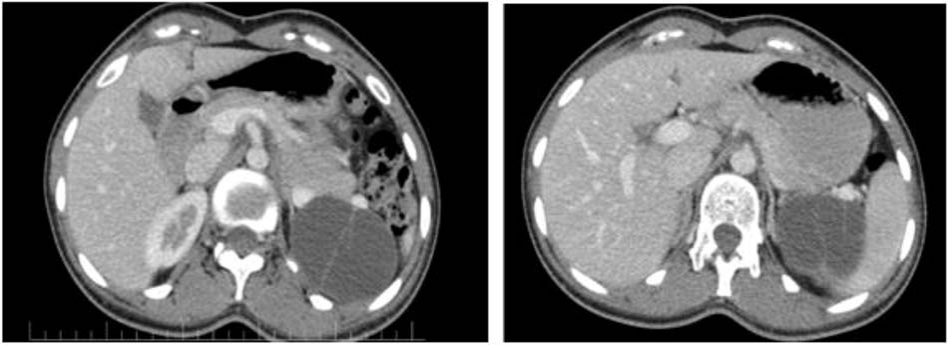
The pancreatic cystic mass appearance on the patient’s CT scan.

On ultrasound, the size, echogenicity, and parenchyma of the liver were normal, the bile ducts within and outside the liver were not dilated. Diameters of the port, bile ducts, and gallbladder were normal. The spleen and pancreas had normal size. And the pancreatic ducts were not dilated. A 61 × 50 mm cystic lesion with internal septa was observed adjacent to the upper bridge of the left kidney. The patient’s abdominal CT showed a clear thin-walled cystic lesion containing septa in the spleen with a size of 50 × 61 × 48 mm (Fig. 1).

**Figure 2:**
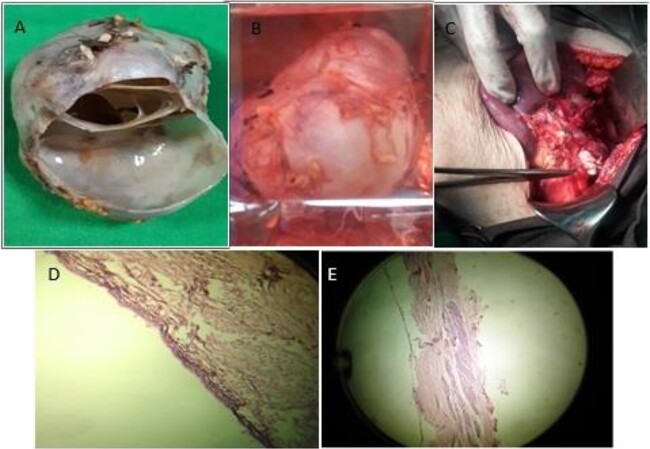
The macroscopic appearance of the pancreatic cystic mass during (**A**) and after surgery (**B**, **C**), and the microscopic appearance of the mass (**D**, **E**).

The patient had a surgical consultation and underwent an abdominal surgery on the day of admission. The abdominal wall was opened by the right subcostal method and distal pancreatectomy was performed by preserving the spleen (Fig. 2B and C). The mass was removed and sent to the pathology for the final diagnostic examination. Macroscopically, the round mass was a markedly milky and soft appearance (Fig. 2A), and microscopically, the cyst wall was marked by a columnar epithelial cell with mucinous cytoplasm and nuclei without atypia in an ovarian stroma (Fig. 2D and E). She did not suffer from any particular complication and was discharged in good condition 4 days after the operation.

## Discussion

The patient was a young woman with nonspecific gastrointestinal symptoms who was diagnosed with MCN after diagnostic procedures. The table below shows the characteristics of patients in previous studies (Table 2).

**Table 2:** Epidemiological characteristics of cystic pancreatic neoplasms in previous studies

Previous studies	Number of patients	Gender frequency (man:woman)	Age average Year	Mass location (H:B-T)^a^	Average size (cm)
Fukushima [7]	9	0:9	49	0:9	8.6
Thompson [8]	130	0:130	45	5:125	10.6
Zamboni [9]	56	0:56	48	4:52	8.4
Hara [10]	5	0:5	54	0:5	9.4
Izumo [11]	34	0:34	44	0:34	8.4
Yamao [12]	15	0:15	45	0:15	8.5
Reddy [5]	56	1:55	48	4:52	5.0
Park [13]	90	1:89	48	6:84	6.5
Scourtas [14]	136	4:132	53	26:110	4

^a^H:B-T, Head: Body-Tail

Women account for 98% of MCN cases and are mainly affected in the fifth decade of life (in 95% of cases, at 47+-15 years of age) [5]. In general, these are solitary and solid tumors that are often located in the trunk and tail of the pancreas and the maximum size of the tumor is in the range of 5 to 19 cm [6].

Clinical appearances are nonspecific and 62% of patients have abdominal pain, 11% weight loss (11% abdominal mass, 9.9% acute pancreatitis, 8.9% fatigue, and 16% without symptoms). Large cystic pancreatic neoplasms of the mucosa can put pressure on the surrounding organs and structures, leading to abdominal pain and a feeling of fullness, but in general, many patients are asymptomatic and the lesions are accidentally seen on imaging [7]. The patient had a history of abdominal pain on the right side with a not-so-favorable response to routine treatments.

MRI has a higher diagnostic accuracy than CT scans in this cyst (19.9 vs. 1.2%). MRI also more accurately defines the association of the pancreatic duct with the lesion and can distinguish cyst-like from IPMN. MRI also improves cyst content in T1 and T2, which is its most important advantage over CT scan [8]. Any pancreatic cyst >1 cm should be examined by abdominal CT scan or abdominal gadolinium MRI. The appearance of MCN is different in several form, it can be very similar to cyst-like or cystic-adenoma serous. It may have one or more walls or it may appear as a very large cyst (>2 cm) [9]. MCNs also account for one-third of cases of invasive cancer [10]. The mass that underwent surgery in this study was a clear, walled cystic lesion measuring 48–50 mm.

Risk factors for malignancy are large lesions, with nodules or masses, calcification, walls >2 cm in thickness, presence of septa, and high patient’s age [11]. Mucinous cystadenocarcinoma is the most common malignancy expected in this type of cyst, which is similar to pancreatic ductal adenocarcinoma. Other types of neoplasms that can occur include undifferentiated carcinomas with giant cells such as osteoclast, adenosquamous carcinoma, choriocarcinoma, and high-grade sarcoma. In all cases, several samples of the mass should be taken because the invasive components are small and can easily be overlooked [12]. If there is malignancy, there is a possibility of liver involvement, peritoneal involvement, and local invasion of the tumor, which need to be investigated and ruled out. In the mass pathology of this patient, there were columnar epithelial cells with mucinous cytoplasm and nuclei without atypia, and no evidence of peritoneal and local invasion was seen.

In 2010, the International Association of Pancreatology recommended the removal of all MCNs for the following reasons: the lack of specificity of diagnostic tests and biopsy specimens, the risk of malignant transformation of 6–27%, the impossibility of distinguishing non-invasive lesions from invasive lesions, and good results of a complete surgery. The association also recommends laparoscopic resection, parenchymal resection, and distal pancreatic resection for lesions <4 cm without mural nodules [13]. The surgery of non-invasive MCN does not require any postoperative diagnostic follow-up, whereas after surgical resection of MCNs with invasive components, diagnostic follow-up (CT/MRI) should be performed every 6 months [14]. In the proposed patient, the surgical treatment method was diagnostic therapy, which was performed distal pancreatectomy with preservation of the spleen for the patient.

## Data Availability

All data (of the patient) generated during this study are included in this published article.

## Conclusion

Pancreatic mucinous cysts are low-prevalent masses that affect most women in their fifth decade of life. But they happen at other ages, as was the case. Depending on the characteristics of the tumor, the symptoms may be very vague and nonspecific. Diagnostic imaging (CT/MRI) is their best diagnostic method, and a complete surgical resection is the only treatment that improves long-term survival of patients.
